# The protective role of professional self-concept and job embeddedness on nurses’ burnout: structural equation modeling

**DOI:** 10.1186/s12912-021-00727-8

**Published:** 2021-10-19

**Authors:** Soghra Goliroshan, Monir Nobahar, Nayyereh Raeisdana, Zahra Ebadinejad, Parvin Aziznejadroshan

**Affiliations:** 1 Student Research Committee, Semnan University of Medical Sciences, Semnan, Iran; 2 Nursing Care Research Center, Semnan University of Medical Sciences, Semnan, Iran; 3 Social Determinants of Health Research Center, Semnan University of Medical Sciences, Semnan, Iran; 4 Faculty of Nursing and Midwifery, Semnan University of Medical Sciences, Semnan, Iran; 5 Nursing Care Research Center, Health Research Institute, Babol University of Medical Sciences, Babol, Iran; 6 Faculty of Nursing and Midwifery, Babol University of Medical Sciences, Babol, Iran

**Keywords:** Burnout, Job embeddedness, Nurse, Professional self-concept

## Abstract

**Background:**

Job embeddedness and professional self-concept are among the important nursing components, the existence of which help decrease occupational burnout among nurses. This study aimed to determine the protective role of Professional Self-concept and Job embeddedness on nurses’burnout.

**Methods:**

This descriptive, correlational study had a predictive design and was conducted on nurses working in training and healthcare centers of Babol University of Medical Sciences in 2019. In total, 308 nurses participated in this study and were selected by stratified random sampling. In addition, data were collected using demographic characteristics questionnaire, Professional Self-concept questionnaire, Job embeddedness scale and nurses’ burnout questionnaire. Moreover, data analysis was performed in SPSS version 25 and Smart PLS version 3.3 using correlational statistics and structural equation modeling.

**Results:**

Both the variables of professional self-concept and job embeddedness, had a significant effect on nurses’ burnout at 99% confidence level (*P* < 0.001) and the negative beta value for these two variables shows the inverse relationship between both professional self-concept and job embeddedness with nurses’ burnout. The value of the coefficient of determination for burnout indicates that both the variables of professional self-concept and job embeddedness, together explain 78% of the changes in the variable of burnout. The beta coefficient for professional self-concept (− 0.50) is higher than the same coefficient for job embeddedness (− 0.42). As a result, the role of professional self-concept in predicting burnout of clinical nurses has been more than the role of job embeddedness. The indirect effect of professional self-concept on burnout of clinical nurses mediated by job embeddedness has been equal to − 0.347. As a result, it can be said that nurses’ professional self-concept has a significant effect on nurses’ burnout through mediation of job embeddedness.

**Conclusion:**

According to the results of the study, Professional Self-concept had an effective role in nurses’ burnout. In other words, the higher the Professional Self-concept of nurses, the lower their burnout. Therefore, it is suggested that effective interventional strategies be designed by nursing managers through better planning and a supportive workplace be established to improve Professional Self-concept among nurses and decrease their burnout.

**Supplementary Information:**

The online version contains supplementary material available at 10.1186/s12912-021-00727-8.

## Background

Nurses face various stresses during their service. There is a close relationship between burnout and stress, hence they are particularly prone to the problem of burnout which may result in decreased quality of care [1–3]. Psychological burnout is comprised of three core dimensions which are emotional exhaustion, depersonalization, and reduced personal accomplishment. Burnout occurs due to imbalance between abilities, skills and available sources with the occupational necessities and demands. Burnout is recognized as a problem in all healthcare systems [4, 5]. In a way that one out of seven nurses experiences exhaustion and burnout at the end of a working day [6]. In addition to its adverse effect on the physical and mental health of the personnel, burnout decreases efficiency, organizational commitment, self-esteem, citizenship behaviors, creativity, innovation, and the quality of nursing services [7, 8]. On the other hand, it can increase hospital costs [6, 9], frustration, disappointment, dissatisfaction [10] and result in deviant, destructive behaviors and turnover. It can also lead to aggression towards patients and co-workers and inability to communicate with family and friends [11].

In a study by Hayes et al. (2015) in Australia, 52.5% of 417 nurses had high emotional exhaustion, whereas 53 and 58% of them had a high level of depersonalization and lack of accomplishment, respectively [12]. In 2018, Biganeh et al. conducted a research in Shahrud, Iran, demonstrating that 40.7% of nurses experienced a moderate level of emotional exhaustion while 62.79 and 68.6% of them had a low level of depersonalization and lack of accomplishment, respectively [13].

Various studies have been conducted on burnout in Iran and most of them are about its prevalence among nurses or investigate its relationship with a component. Since, extensive research is required in this area to recognize the factors related to the burnout of nurses, and a single study cannot determine all effective variables in this regard [14]. It is necessary to conduct further studies on other factors accompanying burnout to take appropriate measures in designing programs to prevent or reduce burnout [10]. One of the variables addressed in the present study was professional self-concept, because very few studies have been done in our country and none of them investigated its relationship with burnout. However, this study demonstrates that professional self-concept can lead to employees’ success in the workplace [15], improvement of individual behaviors and role development [16]. Moreover, a positive professional self-concept can play an important role in enhancing adaptation in the stressful nursing environment [17]. Cao (2010) showed that a positive understanding of professional self-concept can decrease the stress and burnout experiences in clinical nurses [18]. Based on the foregoing issues and considering the effects and complications imposed by burnout on the individual and the organization, the researchers of this study decided to investigate the association between this factor and burnout in nurses.

Professional self-concept means the understanding that nurses have of themselves in the nursing work environment [19]. Nurses’ self-concept reflects the information and beliefs they have about their role, values, and behaviors, and is an attitude that enhances their sense of professional identity [10]. The nurses’ professional self-concept is a developing process which starts as soon as the individual is accepted as a nursing student at the university and continues and develops until the time they are allowed to work as a graduate nurse in the clinical context [20]. Self-concept is one of the important components in health-related professions, especially nursing. Studies have emphasized the necessity of this issue in the nursing profession [21, 22], owing to its considerable role in nurses’ abilities to carry out their professional duties. Nurses with higher professional self-concept are more accountable toward their patients and work results and take care of patients with more respect and interest [17]. Positive professional self-concept improves nurses’ empowerment and clinical performance and problem solving and subsequently enhances the quality of nursing care provided. In contrast, nurses with low professional self-concept have fewer clinical competencies, lower occupational satisfaction and a higher intention of turnover [23]. In a research by Nwafor et al. (2015) in Nigeria, there was a positive relationship between professional self-concept and occupational satisfaction. On the other hand, a negative association was found between professional self-concept and occupational satisfaction with burnout [10]. In 2017, Mosayebi et al. found a negative linear correlation between occupational tension and professional self-concept in Tehran, Iran, in a way that the professional self-concept of nurses increased with the decrease of their occupational tension, and nurses’ occupational tension decreased with the increase of their professional self-concept [17].

Turnover and frequent Nurses displacement between hospital wards are costly problems that healthcare providing organizations face all the time [24]. They always look for a solution to keep their nurses [25] because lack of staff or having unskilled personnel decline the quality of the care provided for the patients. Viewing the concept of Job embeddedness can help in this regard, as this concept provides a structure based on the retention of the employees in their organizations and focuses on examining the reasons for which the employees stay in their jobs with regard to organizational factors and the society [26]. Since only one study investigated this concept in Iran [27] and considering the fact that the evidence shows Job embeddedness can predict the personnel relocation and the absence from work [28] and burnout is one of the most important factors that leads to leaving the job, the researchers of the present study decided to investigate the association between Job embeddedness and burnout and in fact answer whether Job embeddedness can demonstrate burnout or not? Answering this question can pave the way for healthcare managers to reduce or prevent burnout and retain the medical personnel. According to what was said, Job embeddedness shows a new perspective on organizational behavior that can be established and maintained based on the relationships that the employees form with people, organizations and activities inside and outside the organization.

Job embeddedness refers to a collection of forces that connect people to their jobs [29]. These forces are shaped inside and outside a job and refer to forces that influence employee retention. In fact, they are indicative of individuals’ perceptions of their relationship with aspects of work such as people and groups, person-job fit, and self-sacrifice discussed in turnover [30]. The main purpose of Job embeddedness is realizing the reasons for employee retention based on factors related to the organization and community [26]. In a research by Reitz et al. (2011) in the United States, there was a strong conceptual relationship between Job embeddedness and employee retention, in a way that Job embeddedness explained 24.6% of the variance of employee retention [25]. In addition, Job embeddedness encompasses three components of links (close work relationship with colleagues), fit between profession and goals and individual values, and sacrifice in career. Together, these components act as a shield against damages and hardships that might be the precursors of turnover [31]. In 2018, Hopson et al. in the United States concluded that Job embeddedness includes occupational satisfaction and acts as a method to prevent turnover and increase the retention of nurses and is associated with increased age, social communications and group of peers [26, 27]. In Hong Kong, Ng et al. (2009) believed that Job embeddedness had a positive relationship with performance and creativity in the job and a negative association with inefficient behaviors. Different components of Job embeddedness have various effects on job consequences. In this context, embeddedness had a positive effect on the main job performance, communications had a positive effect on creativity, and sacrifice had a positive effect on social behavior [30]. Given the fact that burnout is the most important problem of a profession affecting nursing turnover, and since no research has evaluated the role of self-concept and Job embeddedness on burnout of nurses, this study aimed to determine the protective role of professional self-concept and Job embeddedness on nurses’ burnout.

## Methods

### Aim & Hypotheses

The study aimed to determine the protective role of professional self-concept and Job embeddedness on nurses’ burnout.

Therefore the researchers of this study sought to confirm the following hypotheses:

1. Professional self-concept and job embeddedness have a protective role on burnout of clinical nurses
2. Professional self-concept mediated by job embeddedness has a protective role on burnout of clinical nurses

### Design of the Study

This was a descriptive-correlational study with a predictive design, which was conducted in 2019. The study population consisted of all nurses employed in 4 hospitals (Shahid Beheshti, Shahid yahya nejad, Rouhani and Amirkola) of Babol University of Medical Sciences, Iran. A total of 350 nurses were selected by using sample volume formula with confidence interval of 95%. In the Additional file 1, we explained how to select the samples in all hospitals included in the study (please see the Additional file 1 for more information). From 350 questionnaires that distributed among nurses, 308 complete questionnaires were obtained and the participation rate was 88%. The participants were selected by random sampling. The self-report questionnaires were completed by the nurses. The research instruments were distributed by the researcher in various work shifts and collected after completion.

The inclusion criteria were having a BSc or a higher degree in nursing, a minimum of one year of nursing work experience during the research and willingness to participate in the study. In case any of the participants had a physical or mental illness or did not like to take part in the study, they were not included in the study from the beginning. Moreover, if a questionnaire was incomplete, it was excluded from the study.

### Data collection instruments

Data were collected using the Demographic characteristics questionnaire, Professional Self-concept questionnaire, Job embeddedness scale, and nurses’ burnout questionnaire.

Demographic characteristics included age, gender, level of education, marital status, work experience, shift schedule, employment status, type of ward, and monthly overtime hours. Descriptive statistics of demographic characteristics are mentioned in Table 1.

**Table 1:** Demographic characteristics of participants

Variable	Group	n	%
Hospital	Rohani	129	41.9
	Beheshti	74	24.0
	Amirkola	51	16.6
	Yahya nejad	54	17.5
Gender	Female	244	79
	Male	64	21
Marital status	Single	51	16.6
	Married	256	83.1
	Widow or divorced	1	0.3
Level of education	Bachelors degree	273	88.6
	Master degree	35	11.4
Type of ward	General	189	61.4
	Critical care	119	38.6
Shift schedule	Morning shift	24	7.8
	Circulating	284	92.2
Work experience (Years)	≤5	59	19.2
	6–10	79	25.6
	11–15	99	32.1
	16–20	39	12.7
	21–25	17	5.5
	26–30	15	4.9
Monthly overtime hours	< 50	43	14
	50–100	169	54.9
	150–100	83	26.9
	150<	13	4.2
Employment status	Formal	226	73.3
	By Contract	43	14
	Compulsory service course	39	12.7

Note: *n* = 308

The professional self-concept of nursing instrument is a 36-item tool encompassing six dimensions of general self-concept (self-esteem), communications, knowledge, care, leadership and communication with colleagues. The instrument is scored based on a six-point Likert scale (The Likert scores of this questionnaire were 1–6), and the score range of the tools is 36–216 (6–36 range of score for each dimension) [32]. The questionnaire has been translated into Persian by Badieh Peyma et al. (2013), and Cronbach’s alpha was estimated at 0.89 and 0.97 for reliability of the tool, and for evaluate the validity of the questionnaire, a construct validity (intre-item reliability) and comparison of known groups were used. The range of these coefficients ranged from 0.41–0.75 and all of them were significant [33].

The Job embeddedness scale comprises two subscales of internal (organization or on-the-job) (six items) and external (community or off-the-job) (seven items) Job embeddedness [31]. The scale is scored based on a five-point Likert scale, from completely disagree (one score) to completely agree (five scores). In addition, the score range of the scale is 13–65, and its reliability was estimated at the Cronbach’s alpha of 0.73 (internal embeddedness) and 0.80 (external embeddedness), respectively [34].

On the other hand, the burnout of nurses was assessed by applying the Maslach Burnout Inventory (MBI) (1981) [14]. This 22-item tool includes three dimensions of emotional exhaustion, depersonalization and lack of accomplishment, and the items are scored based on a seven-point Likert scale (never = 0 to very high = 6). While the dimension of emotional exhaustion has nine items and evaluates being tired of working too much, the dimensions of depersonalization and lack of accomplishment include five and eight items assessing the level of indifference and a sense of depersonalization, and feeling competent and successful at work, respectively. In this tool, the high score of emotional exhaustion and depersonalization or the low score of individual accomplishment demonstrate high levels of burnout. In the emotional exhaustion dimension, the cutoff points of < 16, 17–26, and ≥ 27 were low, moderate and high, respectively. In the depersonalization dimension, the cutoff points of < 6, 7–12 and ≥ 13 were low, moderate and high, respectively. Ultimately, the cutoff points of ≤31, 32–38 and ≥ 39 were low, moderate and high, respectively, in the lack of accomplishment dimension. Maslach reported a Cronbach’s alpha of 0.71–0.90 for the three dimensions, and the tool’s reliability was estimated in the range of 60–80 using a re-test [14]. In the present study, the reliability of the tool was confirmed at the Cronbach’s alpha of 0.786.

First, nurses who met the inclusion criteria were selected by referring to various hospitals wards following the obtaining of permissions from the ethics committee of Semnan University of Medical Sciences and from the authorities of hospitals in Babol, Iran. After explaining the research objectives to the subjects and ensuring them of the confidentiality terms regarding their personal information, the questions raised by these individuals were answered and written informed consent was obtained prior to the research. In the next stage, the data collection tools were provided to nurses and were collected one week later.

### Ethical consideration

We adhered to the ethical considerations by obtaining a license from the ethics committee (IR.SEMUMS.REC.1398.041) and the vice-chancellor for research of Semnan University of Medical Sciences, receiving an introduction letter to conduct the study in Babol University of Medical Sciences, explaining the research objectives to the participants and obtaining their consent to participate in the research, ensuring them of the voluntary participation in the study and the confidentiality terms. In addition, we appreciated the cooperation of the subjects and the authorities for their cooperation with the study.

### Data analysis

Descriptive and inferential statistical methods have been used for statistical analysis. In the descriptive part, the characteristics of research variables are expressed and in the inferential part, by using the Structural Equation Model (SEM) and the use of Smart PLS software, the identified research paths in accordance with the conceptual model are examined. Version 3.3 of Smart PLS software has been used to develop a confirmatory factor analysis and structural equations model, and SPSS version 25 has been used for descriptive statistics [35]. It is necessary to mention that due to the multi-level model and the multiplicity of questionnaire indicators, the PLS method has been used.

## Results

In this study, the mean age of the participants was 35.59 ± 7.35 years. Other Demographic characteristics and descriptive statistics of model variables are mentioned in Tables 1 and 2.

**Table 2:** Descriptive statistics of model variables

The main variables of the model	Model components	Descriptive indicators		Normality indicators		Variation range		Level
		Mean	Standard deviation	Skewness	kurtosis	The least	The most	
Burnout	Emotional Exhaustion	32.78	10.69	− 0.33	−0.17	2	54	Sever
	Depersonalization	24.33	5.35	−1.19	1.10	6	30	Sever
	Personal Accomplishment	28.98	6.92	0.18	0.14	7	48	Weak
Professional self-concept	Care	28.14	4.23	−0.41	0.25	13	36	High
	Communications	28.93	4.37	−0.35	0.05	15	36	High
	Knowledge	27.27	4.90	−0.25	−0.40	14	36	High
	Leadership	25.39	4.69	−0.38	−0.05	13	36	High
	Communication with Colleagues	28.46	4.16	−0.38	0.30	14	36	High
	Self-esteem	25.95	5.96	−0.48	0.03	7	36	High
Job embeddedness	Internal (organizational)	22.50	4.16	−0.34	−0.09	10	31	High
	External (community)	23.34	5.29	0.12	−0.21	7	37	High

According to the results, 45.1% (*N* = 139) individuals suffered from burnout. Regarding dimensions of burnout, the highest number of 73.7% of nurses had high Emotional Exhaustion, 95.5% had severe Depersonalization and 67.5% had low levels of Personal Accomplishment.

Before entering the test phase of hypotheses and conceptual model of research, it is necessary to ensure the accuracy of the models for measuring exogenous and endogenous variables. Confirmatory factor analysis method is used to find the underlying variables of a phenomenon or to summarize the data set. To evaluate the validity of the measurement models, we calculate the following values, and if the conditions listed in Table 3 are met, we can claim that the measurement model has the appropriate conditions.

**Table 3:** Conditions for establishing reliability and convergent validity

Index	Acceptable range
Reliability	Composite Reliability and Cronbach’s alpha are above 0.7
Convergent validity	Factor Loads must be significant (t > 1.96)
	Standard factor loads must be greater than 0.4.
	CR > AVE
	AVE > 0.5
	Rho_A > 0.06
	Q^2^ > 0
Divergent validity	AVE > MSV

CR: Composite Reliability, AVE: Average Variance Extracted, VIF: Variance Inflation Factor, MSV: Maximum Shared Squared Variance, Q^2^ Index (Stone-Geisser Index).

According to the results obtained from Table 4, all indicators had a factor load greater than 0.5 and were significant (t > 1. 96) at 95% confidence level. One of the indicators of convergent validity test is the Average Variance Extracted index (AVE), which is actually a percentage of the variance described among the items. As shown in Table 3, the value of this index for model constructs should be higher than 0.5 to indicate convergent validity in the model.

**Table 4:** Convergent validity index, reliability

Main variables	Components	item	Factor loading	T-Value	VIF	Cronbach’s alpha	Rho_A	CR	AVE
Professional self-concept	Care	a1	0.782	29.109	1.953	0.894	0.903	0.915	0.548
		a2	0.774	32.355	2.031				
		a3	0.89	78.234	3.728				
		a4	0.72	22.597	1.828				
		a5	0.888	73.291	3.575				
		a6	0.771	28.967	1.921				
	Communications	b1	0.715	21.661	1.601	0.806	0.807	0.866	0.565
		b2	0.608	12.068	1.345				
		b3	0.697	15.89	1.573				
		b4	0.699	18.714	1.773				
		b5	0.788	32.94	2.429				
		b6	0.782	32.621	2.03				
	Knowledge	c1	0.713	21.099	1.571	0.91	0.911	0.927	0.614
		c2	0.598	11.74	1.539				
		c3	0.772	28.017	2.124				
		c4	0.83	38.642	2.522				
		c5	0.61	5.33	1.447				
		c6	0.807	43.177	2.482				
	Leadership	d1	0.82	34.53	2.22	0.891	0.9	0.917	0.651
		d2	0.822	39.591	2.547				
		d3	0.837	41.822	2.675				
		d4	0.768	24.94	1.895				
		d5	0.861	48.65	2.712				
		d6	0.799	31.176	2.228				
	Communication with colleagues	e1	0.805	35.312	2.117	0.809	0.814	0.863	0.515
		e2	0.755	21.926	2.108				
		e3	0.9	84.03	3.592				
		e4	0.844	43.866	2.559				
		e5	0.823	37.522	2.43				
		e6	0.848	51.757	2.579				
	Self-esteem	f1	0.569	13.124	1.456	0.817	0.836	0.869	0.529
		f2	0.631	14.06	1.589				
		f3	0.735	22.207	1.577				
		f4	0.767	25.206	1.68				
		f5	0.748	22.919	1.675				
		f6	0.686	15.139	1.482				
Job embeddedness	Internal (organizational)	g1	0.772	27.929	2.733	0.901	0.902	0.924	0.67
		g2	0.82	35.28	3.287				
		g3	0.828	38.598	2.426				
		g4	0.844	43.225	2.85				
		g5	0.803	30.368	2.293				
		g6	0.79	29.805	2.198				
		g7	0.759	27.318	2.015				
	External (community)	h1	0.744	25.537	1.842	0.909	0.915	0.93	0.689
		h2	0.745	19.899	2.359				
		h3	0.793	29.084	2.687				
		h4	0.816	43.09	2.141				
		h5	0.757	22.383	1.898				
		h6	0.81	31.155	2.305				
Burnout	Emotional exhaustion	i1	0.75	21.832	1.582	0.783	0.797	0.846	0.548
		i2	0.807	31.655	1.896				
		i3	0.771	27.403	1.618				
		i4	0.761	24.97	1.703				
		i5	0.661	15.613	1.284				
	Depersonalization	j1	0.811	39.128	2.931	0.87	0.876	0.902	0.605
		j2	0.828	41.325	3.077				
		j3	0.79	31.031	2.603				
		j4	0.718	23.908	1.841				
		j5	0.772	29.404	2.316				
		j6	0.521	10.6	1.406				
		j7	0.743	23.267	2.075				
		j8	0.79	33.276	2.461				
		j9	0.637	13.951	1.567				
	Personal Accomplishment	k1	0.805	38.663	2.389	0.908	0.909	0.927	0.644
		k2	0.845	48.506	2.904				
		k3	0.733	22.903	1.855				
		k4	0.749	27.231	2.07				
		k5	0.761	27.425	2.007				
		k6	0.819	39.982	2.442				
		k7	0.793	37.498	2.539				
		k8	0.758	28.94	2.403				

The results of Table 4 indicate that the value of this index for all research variables was higher than 0.5. Another convergent validity index is Rho_A index. The value of this index for all research variables has been higher than 0.06, which indicates the confirmation of convergent validity (Table 4).

To evaluate the reliability of the research variables, two indicators of combined reliability and Cronbach’s alpha have been used. For all research variables, the Cronbach’s alpha value and the composite reliability are greater than 0.7, indicating the reliability of the measurement tool. The Variance Inflation Factor (VIF) index was used to examine the linearity between indicator. All indicators have a VIF value less than 4. Table 5 deals with divergent validity in addition to examining correlation coefficients.

**Table 5:** Correlation coefficients and divergent validity index and descriptive statistics

Model components	Emotional exhaustion	Depersonalization	Personal accomplishment	Care	Communications	Knowledge	Leadership	Communication with colleagues	Self-esteem	Internal	External
Emotional exhaustion	0.74										
Depersonalization	0.433	0.752									
Personal accomplishment	0.57	0.609	0.784								
Care	−0.631	−0.625	−0.549	0.807							
Communications	−0.584	−0.654	−0.475	0.663	0.717						
Knowledge	−0.667	−0.688	− 0.521	0.675	0.374	0.727					
Leadership	−0.453	−0.693	− 0.528	0.589	0.568	0.632	0.818				
Communication with colleagues	−0.523	− 0.473	− 0.648	0.605	0.595	0.667	0.763	0.83			
Self-esteem	−0.525	− 0.484	− 0.425	0.507	0.569	0.609	0.493	0.447	0.763		
Internal (organizational)	−0.645	− 0.614	−0.639	0.591	0.49	0.57	0.584	0.63	0.365	0.778	
External (community)	−0.479	− 0.433	− 0.615	0.571	0.608	0.339	0.428	0.465	0.571	0.672	0.803

*** On the main diameter of the second root is the Average Variance Extracted

Divergent validity is the extent to which a structure is properly distinguished from other structures by empirical criteria. This validity is calculated at two levels of observed variable and latent variable. At the level of the observed variable, to calculate the divergent validity, cross-loads are used. The load of one observed variable corresponding to the structure must be more than all the loads of that variable observed on other structures. At the latent variable level, the Fornell-Larker criterion (Table 5) was used [36]. The second root of the AVE, each latent variable must be greater than the highest correlation of that construct with other construct in the model. That is, the square root of the AVE of the latent variables in the present study, which are located in the cells located in the main diameter of the matrix, is greater than the value of the correlation between them arranged in the lower and left cells of the original diameter. The logic of this construct is that a construct should have more variance with its representations than other constructs [36]. For example, the second root is the AVE for the emotional exhaustion variable (74%), which is greater than the correlation value of this variable with other variables. As shown in the table, the value of the second root of the AVE index, for all variables, is greater than the correlation of that variable with other variables.

**Fig. 1:**
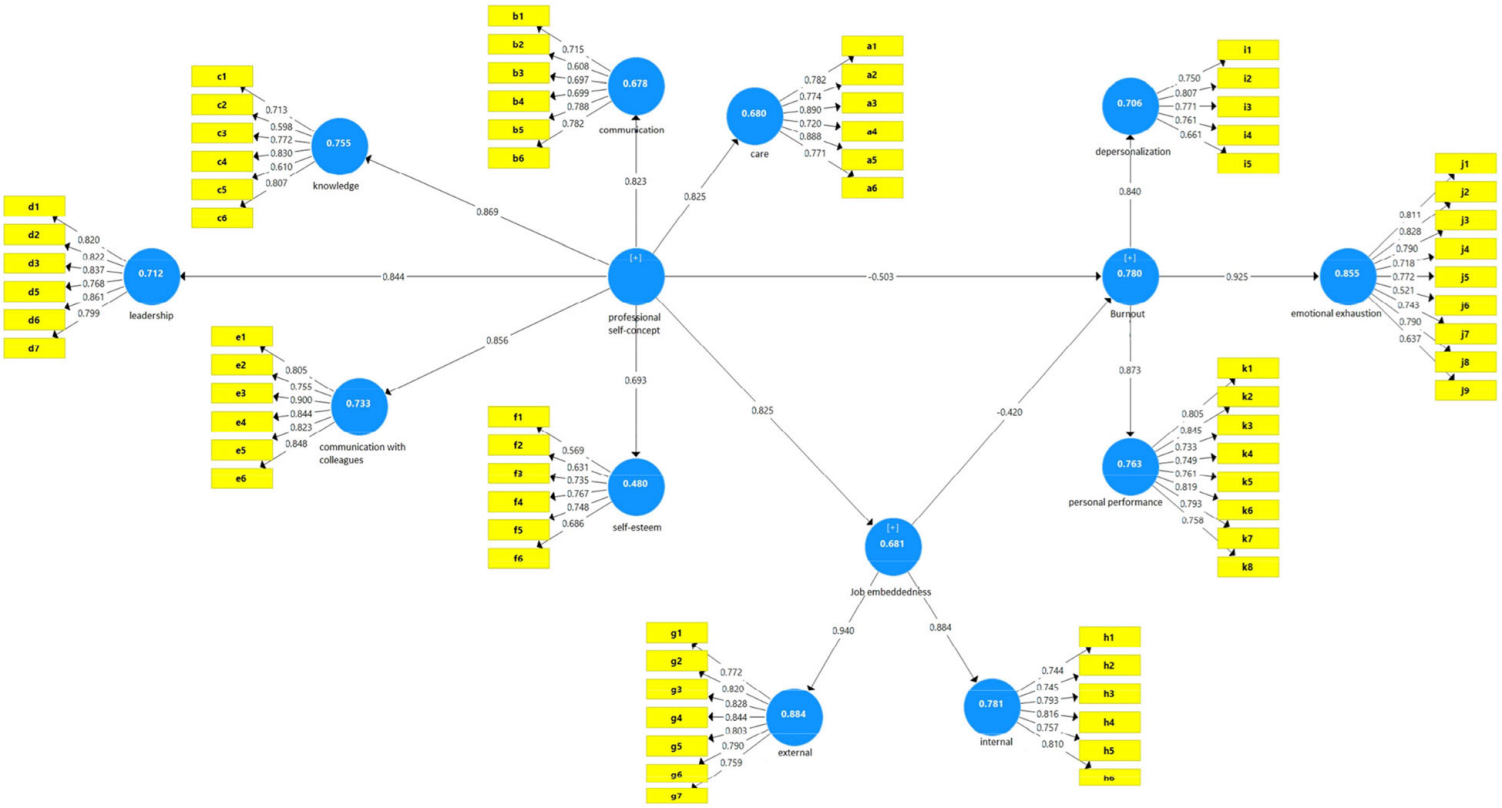
Research model in the state of estimating standard coefficients

Figure 1 shows the structural equation model in the standard coefficient estimation mode. The research model consists of an independent variable called professional self-concept, a mediating variable called job embeddedness and a dependent variable called burnout.

Relationships between circles (latent variables) and rectangles (observed variables) are factor loads. In this diagram, numbers or coefficients are divided into two categories. The first category is called measurement equations, which are the relationships between latent and observed variables (relationships between a circle and a rectangle), these equations are called factor loads. The second category is structural equations that are the relationships between latent and latent variables (relationships between independent and dependent variables). These coefficients are called path coefficients.

**Fig. 2:**
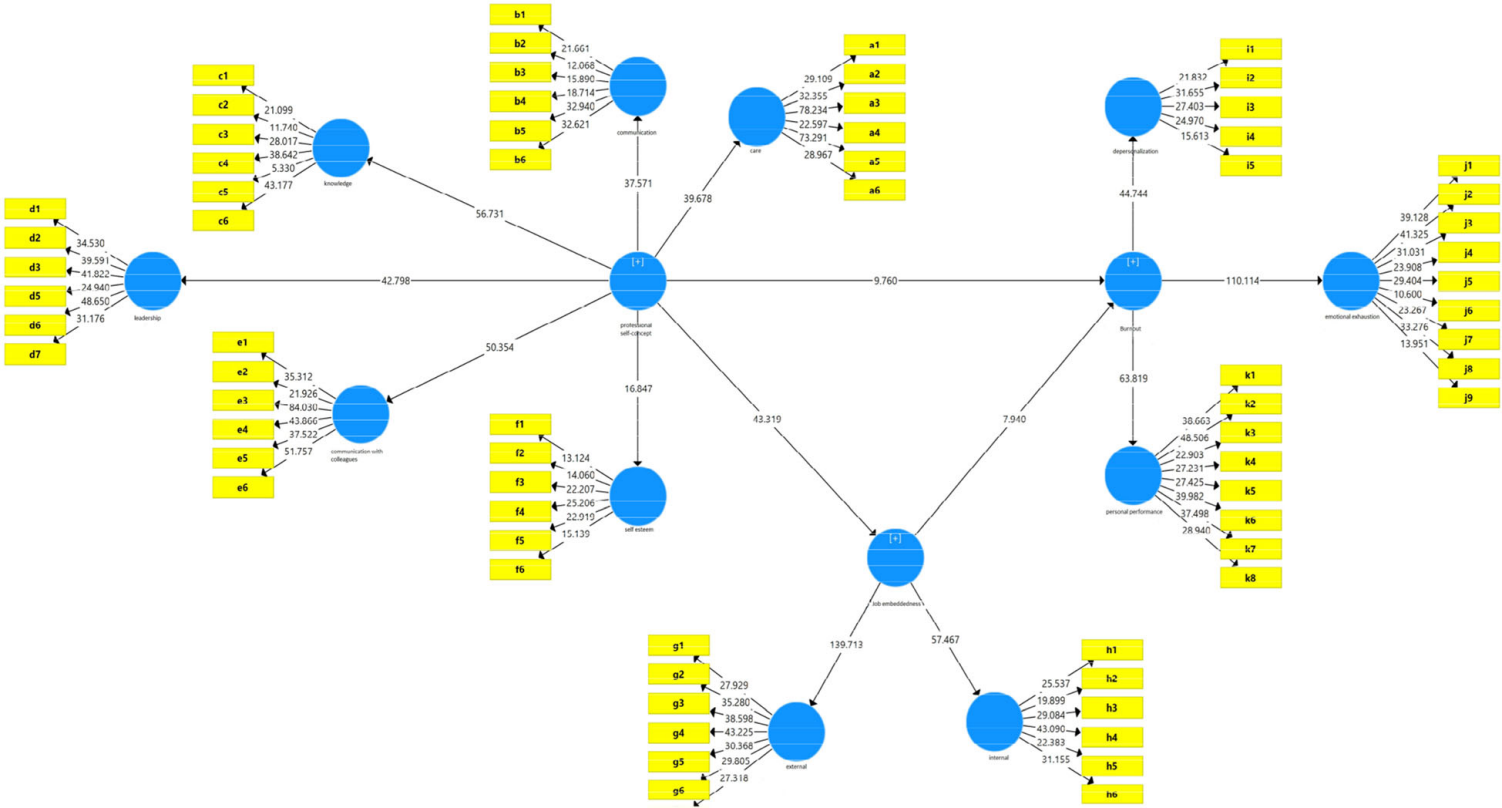
Research model in a significant coefficients

Figure 2 shows the model of structural equations in a significant state.

This model actually tests all measurement equations (factor loads) using the T-value. According to this model, the factor load and path coefficient are significant at the 95% confidence level if the T-value is outside the range of −1.96 to + 1.96.

The results obtained from structural equations show that the direct effect of professional self-concept on job embeddedness is confirmed at 99% confidence level (*P* < 0.001). positive beta value in Table 6 indicates that the relationship between the two variables is positive.

**Table 6:** Path coefficients (beta), T-value, coefficient of determination and result of research hypotheses

Effects	Hypotheses	Beta	T	Significance level	R^2^	R^2^_adj_	F2	Hypothesstatus	Direction
Direct	Professional self-concept- > Job embeddedness	0.825	43.319	0.001	0.681	0.680	–	Confirm	+
	Job embeddedness- > Burnout	−0.42	−7.94	0.001	0.780	0.778	0.256	Confirm	–
	Professional self-concept- > Burnout	−0.503	−9.76	0.001			0.367	Confirm	–
Indirect	Professional self-concept- > Job embeddedness- > Burnout	− 0.347	−7.756	0.001			–	Confirm	–
Control variables	Gender →Burnout	0.098	1.632	0.104	0.056	0.024		Reject	Does not mean
	Age→Burnout	0.039	0.314	0.754				Reject	Does not mean
	Marital status → Burnout	0.032	0.526	0.599				Reject	Does not mean
	Hospital→Burnout	0.100	1.701	0.090				Reject	Does not mean
	Type of ward → Burnout	0.118	2.014	0.045				Reject	Does not mean
	Shift schedule → Burnout	−0.095	−1.488	0.138				Reject	Does not mean
	Level of education →Burnout	0.017	0.297	0.766				Reject	Does not mean
	Work experience → Burnout	0.009	0.069	0.945				Reject	Does not mean
	Employment status →Burnout	0.021	0.372	0.710				Reject	Does not mean
	Monthly overtime hours→Burnout	−0.079	−1.253	0.211				Reject	Does not mean

|t| > 1.96 Significant at *P* < 0.05, |t| > 2.58 Significant at *P* < 0.01.

The index f2 is the effect size. Used when more than one variable affects the dependent variable.

Both job embeddedness and professional self-concept variables had a significant effect on nurses’ burnout at 99% confidence level (*P* < 0.001(and the negative beta value for these two variables shows the inverse relationship between both of them with nurses’ burnout. The value of the coefficient of determination for burnout indicates that both the variables job embeddedness and professional self-concept together explain 78% of the changes in the variable of burnout. The value of beta coefficient for professional self-concept (− 0.503) was higher than the same coefficient for job embeddedness (− 0.42) and as a result, the share of professional self-concept in predicting burnout of clinical nurses was higher than the share of job embeddedness. The indirect effect of professional‌ self-concept on burnout of clinical nurses mediated by job embeddedness has been equal to − 0.347 and this value has been significant at 99% confidence level. As a result, it can be said that nurses ‘professional self-concept has a significant effect on nurses’ burnout through mediation of job embeddedness. Also findings showed that between two dimensions of Job embeddedness, the external (community or off-the-job) dimension has the highest factor load (factor load = 0.94) on this variable, That is, the external dimension of job embeddedness played a more prominent role in the forming of job embeddedness, and the dimension of knowledge (factor load = 0.86) had the most explanatory power of professional self-concept among other dimensions and among the dimensions of burnout, the dimension of emotional exhaustion had the highest factor load (0.92) among the dimensions of MBI and the lowest factor load (0.84) was obtained from the dimension of depersonalization.

Demographic variables are included in the model as control variables to investigate their effect on burnout. None of the control variables including gender, age, marital status, hospital, ward, shift work, education, work experience, employment status and overtime had a significant effect on burnout at 95% confidence level (*P* > 0/05).

## Discussion

According to the results, hypothesis 1 of the study, which was the protective role of professional self-concept and job embeddedness on nurses’ burnout, was supported. The Professional Self-concept and job embeddedness had a direct, negative and significant effect on nurses’ burnout. In other words, burnout of nurses decreased with an increase in their Professional Self-concept and job embeddedness. In this regard, our findings are in line with the results obtained by Coa et al. (2015) in China, and Yu et al.(2019) in south korea, who reported that Professional Self-concept was a significant and negative predictor of burnout. They marked that the increase in Professional Self-concept can decrease burnout [37, 38]. In addition, the results of Wang et al. (2019) in China were indicative of a significant, negative association between the Professional Self-concept of nursing students and their educational burnout. Furthermore, their results supported the stress and coping theory, which states that Pofessional self-concept can affect the stress-related reactions of individuals as a cognitive assessment variable [20]. In this context, Mosayebi et al. (2017) found a negative linear correlation between job stress and Professional self-concept of nurses in Tehran, in a way that increase of job stress was associated with decreased Professional Self-concept [17]. Our results indicate that there is a significant negative correlation between professional self-concept and burnout, and states that Strategies that increase professional self-concept may reduce burnout in clinical nurses. Strategies such as improving nursing knowledge and skills, the ability to communicate and collaborate, self-confidence and sense of leadership are very important for increasing nurses’ self-concept. This finding can light the way for health authorities of the country, especially the nursing organization, to alleviate the shortcomings and improve the situation of nurses’ community by promoting their professional self-concept.

Hypotheses one and two of study that have a direct and mediated impact on job embeddedness on nurses’ burnout was supported. Although it has been reported in several studies that Job embeddedness is an effective factor in employee retention [39, 40], for the protective role of Job embeddedness on nurses’ burnout, unfortunately, the researchers of the present study did not find any study which investigated the relationship between Job embeddedness and burnout in nurses. But they founded a research conducted by Candan (2016), he demonstrated a negative and significant relationship between some dimensions of Job embeddedness and burnout of employees in a state university in Turkey, which is somewhat in line with our findings [34]. Our findings showed that among the dimensions of Job embeddedness, the external (community or off-the-job) dimension has the highest factor load (factor load = 0.94) on this variable, which was contrary to Candan’s findings. He found that the internal (organizational or on-the-job) dimension of Job embeddedness was correltion with burnout. Job embeddedness narrates to organizational-related and community- related reasons as to why people remain in their jobs. Also Albalas et al. (2019) showed that it is vital to improve university staff’s job satisfaction and on-the-job (commitment) dimension to enhance intent to stay at work [41]. Since the job embeddedness theory is focused on reasons why employees stay in a job, it makes sense that programs to improve community fit and community links will help to increase the retention of these employees. As mentioned earlier, Job embeddedness is achieved through a combination of internal and external forces, and the combination and balance of these forces prevents a person from leaving the job [26]. The interest in the nature of the profession and the inter-professional communication are of great importance in the dimension of on-the-job embeddedness (internal job embeddedness). If the forces on-the- job prevail and there are no sources of pressure outside, so person may stay in her/his profession, but if the forces off- the-job (external job embeddedness) increase and the factors on- the- job are not enough to keep the person at profession, so the person will leave it. Therefore, it should be noted that Job embeddedness is not always a positive feature in employees of an organization [42]. Reitz (2010) wrote in the United States that employee retention might occur out of desperation or due to limited employment options while there is negative Job embeddedness [25]. According to this, the different results of our research due to the different conditions prevailing in our field, because the incorrect policies of the Ministry of Health, along with the unfavorable working conditions for nurses and the conflict between work and life and increase family pressure to leave the profession, are among the problems that our nursing community faces. As a result of these factors, nurses have no incentive to stay in the profession other than a source of income, and thus off- the- job embeddedness has become more prominent and tendency to leave the profession and go for informal work increases, therefore we see that due to insufficient sources of income for nurses, some seek Virtual Businesse on the Internet and do a second job in addition to their nursing profession.

According to the results of the present study, the dimension of knowledge (factor load = 0.86) had the most explanatory power of professional self-concept among other dimensions. Therefore, this result confirms that the knowledge dimension played a more prominent role in the forming of nurses’ self-concept. This finding is in line with the results of Karimi et al. (2019) and Yu et al. (2019), They wrote, the dimension of knowledge of professional self-concept is one of the important factors affecting the values ​​of nursing, professional education and learning promotion [38, 43]. Professional self-concept is formed as a result of the relationship between biological and environmental factors and as a result of education and training. It can be concluded that Professional self-concept develops as a result of interaction between humans and environmental factors. Biological and environmental along with education are effective in the formation of professional self-concept, and learning and training have an important role in the development of professional self-concept [43]. Therefore, this issue requires special attention of educational officials and managers and policymakers in the nursing profession to improve the situation. When one’s profession matches one’s self-concept, person sees one’s profession as a meaningful and valuable activity and they will be more successful in the field of patient care and will have a better professional image of themselves [44].

In MBI, mean scores of dimension is indicative of sever burnout. The researches in Iran with regard to nurses’ burnout show that burnout is a common phenomenon in this profession and factors such as low salaries and the poor social identity of this profession, doctor-oriented atmosphere of hospitals, work overload, low job satisfaction, poor authorities’ support and emotional intelligence, are associated with burnout [1, 2, 8, 11]. The dimension of emotional exhaustion had the highest factor load (0.92) among the dimensions of MBI, meaning that the mentioned dimension played the most role in the formation of burnout in nurses. High emotional exhaustion factor load is an important, because it is considered the main core element in burnout and a serious indicator to reach high levels of burnout among nurses. This finding is in line with the results of previous studies [1, 37]. Also according to the results obtained by Hayes et al. (2015) in New Zealand, 52.5% of nurses had severe emotional exhaustion, whereas 53% of the subjects had severe depersonalization [12]. The high level of emotional exhaustion in the present study might be due to the high number of patients, lack of organizational support, high workload in the hospital, low schedule flexibility, dissatisfaction from work environment, inappropriate relationship between manager and the staffs, occupational stresses, and take care of patients with complicated physical problems.

The lowest factor load (0.84) was obtained from the dimension of depersonalization among the dimensions of MBI. This finding is in line with the results of Khazaei et al. (2006), Cao et al. (2015), Soleimani et al. (2015), Who reported that high levels of depersonalization [3, 11, 37]. When people work in areas where there is no proper encouragement, no sense of efficiency and self-discovery, role conflict, low autonomy, negative team relationship [45], rules and policies are not explained, new and diverse approaches are not seen, there are no necessary conditions in order to create peace of mind, people lose their human perspective on patient care while feeling burnout [1, 4]. As the feeling of emotional exhaustion persists, one’s mental capacity decreases; So that he suffers from a kind of cold with extreme indifference to the client and the profession. Hence, depersonalization can be considered a way to adapt to emotional exhaustion. Decreased self-confidence, lack of organizational responsibilities, increased relocation and leaving the profession are the consequences of feeling emotionally exhaustion and depersonalized [8, 9]. Following the feeling of burnout, the quality of patient care decreases and this issue will cause torment of conscience in the nurse and increase in the severity of burnout, and this defective cycle continues [45].

### Limitations of the study

One of the limitations of the present study was that it was a cross-sectional study which was not able to identify the causal relationships between the research variables. Therefore, it is suggested that a longitudinal study be conducted in the future to determine the causal relationships between these variables. Moreover, the self-report method may not adequately reflect the mental behaviors and processes and the actual amount of burnout might be greater than what the nurses have reported. As a result, it is recommended to nursing managers and associations to pay more attention to the researches done in this regard and provide a practical solution to this problem. Another limitation of this study was the geographical restriction which means it examined the samples of only one city. It is suggested that further researches be conducted on this subject with larger and more diverse samples and in a wider geographical area. Given the protective role of professional self-concept of nurses in their burnout, it is suggested that measures be taken so that nurses could be able to achieve the self-concept skills since it would help them gain self-knowledge and assess their abilities and strengths against their job demands. This process could act as a buffer even in difficult working conditions. Since burnout is a multidimensional issue and can be affected by various factors, it is recommended that other factors affecting burnout (e.g., work shift system, workload, sleep disorders and psychological diseases) be assessed in future studies.

## Conclusion

According to the results of the present study, 45% of nurses suffered from burnout. Therefore, designing measures by metrons, such as improving the friendly relations among employees, motivating group work, including employees in decision-makings, giving occupational supports, reducing job conflicts and considering individual differences, could be beneficial in the decrease of burnout. These issues should be considered by nursing managers and authorities at different levels of supervision for the development of physical and mental health of nurses. Nursing managers can also take steps to reduce burnout through strategies such as introducing novice nurses to the hospital environment through clinical coaching, employing support groups, enhancing access to professional development and increasing resiliency among nurses. The present study led to the recognition of the protective role of Professional Self-concept in nurses’ burnout. Our results also demonstrated that the dimension of communications had the most effect among the dimensions of Professional Self-concept. Since teaching communication skills is a cost-effective and effective method for the decrease of nurses’ burnout, it is suggested that proper strategies be used by managers to deal with this issue.

## Supplementary Information

**Additional file 1.** In the Additional file 1, we explained how to select the samples in all hospitals included in the study.

## Data Availability

The datasets generated and analyzed during the current study are not publicly available due to an agreement with the participants on the confidentiality of the data but are available from the corresponding author on reasonable request.

## Abbreviations

MBI: Maslach Burnout Inventory
SEM: Structural Equation Modeling
CR: Composite Reliability
AVE: Average Variance Extracted
VIF: Variance Inflation Factor
MSV: Maximum Shared Squared Variance
