# Supplementary material for: The protective role of professional self-concept and job embeddedness on nurses’ burnout: structural equation modeling
Source: BMC Nurs. 2021 Oct 19;20:203. doi: 10.1186/s12912-021-00727-8 (PMC8524863; doi:10.1186/s12912-021-00727-8)
Supplement: Supplementary file 1 — Additional file 1. In the Additional file 1, we explained how to select the samples in all hospitals included in the study. [file 12912_2021_727_MOESM1_ESM.doc]

**Additional file 1. Detailed numbers of study population and selected sample in all hospitals included in the study**

| Name of hospital | Type of hospital | Total nurses in hospital* | Sample size (nurses surveyed) | Sample Size % | Nurses responded | Response rate % |
| --- | --- | --- | --- | --- | --- | --- |
| Rohani | Public | 480 | 151 | 14.6 | 129 | 85.4 |
| Beheshti | Public | 255 | 80 | 7.5 | 74 | 92.5 |
| Amirkola | Public | 140 | 54 | 5.6 | 51 | 94.4 |
| Yahya nejad | Public | 204 | 65 | 17 | 54 | 83.0 |
| *Total* |  | 1110 | 350 | 12.0 | 308 | 88.0 |

*Estimated numbers
